# *Ocimum basilicum* L. (basil) presents pro-apoptotic activity in an Ehrlich’s experimental tumor murine model

**DOI:** 10.1590/acb393924

**Published:** 2024-07-29

**Authors:** Phelipe Gabriel dos Santos Sant’Ana, William Gustavo Lima, Gabriela Francine Martins Lopes, Sabrina Elisa de Oliveira, Guilherme Augusto Ferreira da Costa, Luciana Alves Rodrigues dos Santos Lima, Elisângela Elduina Ferreira, Ivan Carlos dos Santos, Laila Cristina Moreira Damázio, Rosy Iara Maciel Azambuja Ribeiro, Flávia Carmo Horta Pinto

**Affiliations:** 1 Universidade Federal de São João del-Rei – Departamento de Medicina – Laboratório de Patologia Experimental – Divinópolis (MG) – Brazil.; 2 Universidade Federal de Minas Gerais – Faculdade de Farmácia – Belo Horizonte (MG) – Brazil.; 3 Universidade Federal de São João del-Rei – Departamento de Ciências Naturais – Laboratório de Patologia Experimental – São João del-Rei (MG) – Brazil.; 4 Universidade Federal de São João del-Rei – Departamento de Farmácia – Laboratório de Fitoquímica – Divinópolis (MG) – Brazil.; 5 Universidade Federal de São João del-Rei – Departamento de Engenharia de Biossistemas – Laboratório de Biomoléculas e Biofármacos – São João del-Rei (MG) – Brazil.; 6 Universidade Federal de São João del-Rei – Departamento de Medicina – Laboratório de Reabilitação – São João del-Rei (MG) – Brazil.

**Keywords:** Ocimum basilicum, Apoptosis, Breast Neoplasms

## Abstract

**Purpose:**

This study aimed to evaluate the therapeutic effect of an ethanol extract of *Ocimum basilicum* L. (EEOb) aerial parts against Ehrlich’s experimental tumor (EET) in mice.

**Methods:**

Swiss mice were divided into two groups (control and treated; n = 6). On day 21, all mice were inoculated subcutaneously with 2 × 10^6^ (0.05 mL) EET cells in the left paw for solid tumor development. This study lasted 28 days. Treatment began 24 hours after inoculation with EET. Measurements of dorsoplantar thickness were used to assess tumor growth. The paw pad was collected for histopathological analysis and stained using the argyrophilic nucleolar organizing regions (AgNOR) technique and immunohistochemistry for proliferating cell nuclear antigen, Bcl-2 and Bax.

**Results:**

The treatment of animals with EEOb at 100 mg/kg intraperitoneally was able to reduce the growth (Control = 3.7 ± 0.1 mm vs. EEOb = 5.7 ± 0.2 mm) and the number of AgNORs of solid Ehrlich tumor. The antitumor effect of EEOb was associated with the induction of apoptosis of tumoral cell, as suggested by the reduction of the content of Bcl-2 induced by extract.

**Conclusions:**

The study demonstrated that daily administration of EEOb is able to reduce the growth of EET by induce apoptosis of tumoral cells.

## Introduction

*Ocimum basilicum* L. (basil) (Lamiaceae) is an aromatic spice with a unique flavor whose leaves, dried or fresh, are commonly used in culinary applications worldwide^1^^,^ 2. In addition to culinary applications, basil also has been widely used in folk medicine to treat a wide range of diseases such as headache, cough, diarrhea, dyspepsia, flatulence, gastritis, renal disfunction (as a diuretic agent), and diabetes^3^. In this regard, many studies have confirmed numerous pharmacological properties of *O. basilicum* by pre-clinical and clinical trials^4^^–^ 6.

*In-vitro* studies have demonstrated that ethanol extracts from aerial parts of *O. basilicum* are active against several humans tumor cells such as cervical cancer (HeLa)^6^^,^ 7, breast cancer (MCF-7, T47D and MDA-MB-231)^6^^–^ 8, acute T cell leukemia (Jurkat)^7^, erythroleukemia (K562)^6^, colorectal adenocarcinoma (HT-29 and HCT_116_)^7^^,^ 9, pancreatic cancer (MIAPaCa-2)^7^, prostate cancer (PC3)^6^, and melanoma (B_16_F_10_ cells)^10^. The antitumoral effects were attributed to the phenolic flavonoids and tannins compounds frequently found in *O. basilicum* extracts^6^^,^ 7 ^,^ 9 ^,^ 11.

However, despite the dense body of evidence related to the antitumor activity of *O. basilicum in vitro*, little is known about the effect of this medicinal plant in solid tumor models *in vivo* 11 ^,^ 12. Ehrlich experimental tumor (EET) is a primitive undifferentiated mice breast carcinoma that grows in several murine strains in solid form when inoculated subcutaneously^13^. It presents many similarities with human tumors and is sensitive to anticancer drugs, thus making the EET a valuable tool in exploring the effect of potential cancer chemicals and drugs^13^^–^ 16. Therefore, the aim of present study was to evaluate the antitumoral activity of ethanol extract of *O. basilicum* L. (basil) aerial parts against EET in mice.

## Methods

### Preparation of ethanol extract of *Ocimum basilicum* L. (basil) aerial parts

Plants aerial parts of *O. basilicum* species were collected in Carmópolis de Minas, Minas Gerais, Brazil. An exsiccate was banked at the Herbarium of the Institute of Biological Sciences, Universidade Federal de Minas Gerais, with the register number BHCB 147240. To prepare ethanol extract, 110.8 g of fresh leaves of *O. basicilum* was dissolved in 1,000-mL ethanol 70% v/v in laboratory condition for 10 days. This material was filtered and dried using a rotary evaporator (Fisatom 803; Uberlândia, MG, Brazil), and the yield of these procedure was 5,56 g of ethanol extract. These dried powders were resuspended in saline (NaCl 0,9%) and used in the treatment group.

### Animals

Six-week-old male Swiss mice (Central Vivarium of the Universidade Federal de São João del-Rei, *Campus* Dom Bosco, São João Del-Rei, MG, Brazil), weighing 30 to 40 g, were used in this study. The animals were kept with solid chow and water *ad libitum* in an environment with a controlled temperature of 25 ± 2°C, 40% humidity, and a 12/12-h light/dark cycle.

All experimental procedures strictly followed the international protocols for laboratory animal management, and the methods were approved by the Laboratory Animal Research Ethics Committee of the Universidade Federal de São João del-Rei (protocol nº 033/2012).

The animals were divided into two groups (i.e., control and treated) with six animals each, housed in polycarbonate cages (40 × 45 × 25 cm) and submitted to a 10-day adaptation period prior to the start of the experiment.

### Preparation and inoculation of Ehrlich experimental tumor

The EET cells were maintained in the ascitic form by weekly inoculations with 1 × 10^6^ cells in Swiss mice intraperitoneally (i.p.)^17^. For the experimental procedures, 3 mL of the ascitic fluid were removed, centrifuged (3 min, 2,000 g), and the supernatant was removed. The EET cells were washed three times in saline, counted in the Neubauer’s chamber, and the viability was determined by the trypan blue dye exclusion staining. On the 21st day, all mice were inoculated subcutaneously with 2 × 10^6^ (0.05 mL) EET cells in the left footpad for the development of the solid tumor^18^.

### Treatment of animals with ethanol extract of *Ocimum basilicum* L.

All the animals of control and treated group received daily, through intraperitoneal injection (i.p.), 0.05 mL of saline and 0.05 mL of ethanol extract of *O. basilicum* L. (EEOb) at 100 mg/kg, respectively, until the end of the experiment. These study lasted 28 days. The treatment started 24 h after the inoculation of EET.

### Tumor growth evaluation

For the tumor growth curve evaluation, measurements (duplicate) of the left hind limb dorsal-plantar thickness were carried out. This measurement was performed with a digital caliper immediately before and after the inoculation of the tumor, three times a week, for 28 days. The mean values obtained were considered as the value of the tumor growth of the day^19^.

### Histochemical

Animals were euthanized with anesthesia overdose (100 mg/kg ketamine + 12 mg/kg xylazine, i.p.), and the left footpad was collected for histopathological analysis. Tissues samples were fixed in neutral 10% buffered formalin (pH 7.2) at room temperature. After fixation, tissues were dehydrated through graded alcohol solutions, and embedded in paraffin. Sections (4-μm thickness) were stained using the argyrophilic nucleolar organizer regions (AgNOR) technique^20^^–^ 22. Next, AgNORs were counted using a digital image analyzer (KS300 Program; version 2.0, Kontron Elektronik; London, United Kingdom) from images generated by a Zeiss Axiolab microscope connected to a camera interconnected to a board computer scanner. A total of 30 cell nucleus per case, selected at random in slide, were analyzed at a 1,000x magnification.

### Immunohistochemistry

To evaluate the proliferative activity of the cells, immunohistochemistry for proliferating cell nuclear antigen (PCNA) was performed. In order to evaluate the balance between pro and anti-apoptotic factors in the tissue, Bcl-2 and Bax were marked using the streptavidin–biotin-peroxidase method^23^, allowing insights into the mechanisms related to the survival and resistance of cancer cells to treatment. Tissue sections (4-µm thick) were fixed in formalin and embedded in paraffin and mounted on poly-l-lysine-coated microscope slides. Tumor sections were deparaffinized and rehydrated through xylene and graded alcohols. After antigen retrieval, endogenous peroxidase was blocked (30 min to PCNA and 10 min to Bax/Blc-2) with 3% (v/v) hydrogen peroxide in darkroom and washed in phosphate-buffered saline (PBS). Sections were incubated overnight (4° C) with primary anti-Blc-2 (Santa Cruz Biotechnology, Santa Cruz, CA, United States of America; 1:50), anti-Bax (Santa Cruz Biotechnology, Santa Cruz, CA, United States of America; 1:200), and anti-PCNA (Santa Cruz Biotechnology, Santa Cruz, CA, United States of America; 1:400) antibodies diluted in PBS plus bovine serum albumin (PBS-BSA). The slides were then incubated with secondary antibody (Santa Cruz Biotechnology, Santa Cruz, CA United States of America; 1:200) associated with dextran polymer by 30 min in room temperature. After washing, the slides were incubated with avidin-biotin-horseradish peroxidase conjugate (Strep ABC complex by Vectastain® ABC reagent and peroxidase substrate solution) for 30 min. Both biomarkers ware visualized with the chromogen 3,3’-diaminobenzidine (DAB). Slides were counterstained with Harry’s hematoxylin, dehydrated in a graded alcohol series, cleared in xylene, and coverslipped.

The images of the slides were acquired using an optical microscope and the Axion Vision software LE 4.8.2.0 (London, United Kingdom). For PCNA detection, photographs were recorded at 400x magnification, and the labeling frequency was determined by the percentage of positive cells in 1,000 tumor cells^24^. For analysis of Bax and Bcl-2, ten photos were recorded, and a limit of 10% of positive tumor cells with distinct cytoplasmic pattern was considered positive for Bcl-2 and Bax. The criteria for interpreting Bcl-2 and Bax were:

- Negative score (0): 0%;
- Score 1: between 1–10% positive cells;
- Score 2: between 10–50% positive cells;
- Score 3: more than 50% of positive cells^25^^,^ 26.

### Statistical analysis

Data normality was assessed by the Shapiro-Wilk’s test. Statistical procedures were performed using the GraphPad Prism 5 (GraphPad Software, CA, United States of America) and the unpaired Mann-Whitney’s test, with a 5%-significance level. Data were expressed as the mean ± standard deviation.

## Results and Discussion

*Ocimum basilicum* is a plant used in folk medicine due to its numerous therapeutic properties. Among them, its antitumor effect stands out^3^. Pre-clinical studies have already shown that extracts and fractions obtained from the leaves and other aerial parts of *O. basilicum* have recognized antiproliferative and pro-apoptotic activity against numerous human cell types of solid and hematological cancer^6^^–^ 8 ^,^ 11. However, the scope of evidence regarding the *in-vivo* antitumoral effect of this medicinal plant is very scarce. Thus, in this study we aimed to evaluate the antitumoral activity of EEOb *in vivo* using a solid Ehrlich tumor murine model.

**Figure 1:**
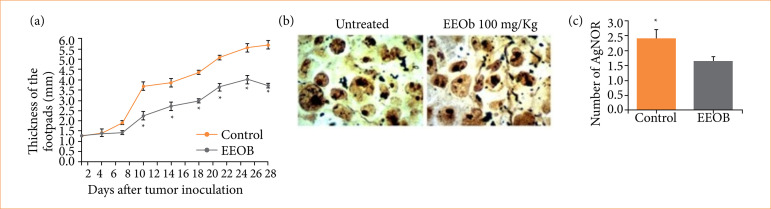
Antitumoral activity of ethanol extract of *Ocimum basilicum* L. (basil) (EEOb) aerial parts against Ehrlich experimental tumor in vivo. **(a)** Tumor growth curve in mice footpads for 28 days. **(b)** Photomicrographs show the subcutaneous tissue of the implantation the Ehrlich tumor stained using the argyrophilic nucleolar organizer regions (AgNOR) technique (1,000X magnification). **(c)** Number the AgNORs of mice footpads inoculated with EET after 28 days. Control group received daily saline solution. EEOb group received daily basil extract at 100 mg/kg intraperitoneally. Data are mean ± standard deviation (n = 6).

Source: Elaborated by the authors.*p < 0.05.

As showed in Fig. 1a, the intraperitoneal use of EEOb at 100 mg/kg significantly reduces the tumor growth in animals (3.7 ± 0.1 mm) compared to the untreated group (5.7 ± 0.2 mm; p < 0.05). For animals that received EEOb, the footpad thickness was significantly reduced from the sixth day to the end of the 28th day of the experiment. Similarly, other studies showed that the oral use of 50% alcoholic aqueous extract of *O. basilicum* (200 mg/kg, p.o.) resulted in significant reduction in tumor volume and lethality in a murine melanoma model (B_16_F_10_ cells)^11^. 

Antitumoral activity of *O. basilicum* can be attributed to some compounds such as polyphenols, hydroxycinnamic acids (caffeic acid, coumarin acid, and rosmarinic acid), flavonoids, and volatile compounds (terpenoids, carotenoids, phenylpropanoids/benzenoids, fatty acid derivatives, and C5-branched compounds)^27^^–^ 29. The chemical composition of volatile compounds of EEOb employed in this study was investigated previously by Araújo et al.^30^ using gas chromatography/mass spectrometry (GC/MS). The major compounds of EEOb were 9,12,15-octadecatrienoic acid ethyl ester (26%), phytol (18%), and cadinene (11%). The phytol has inhibitory effects on the pro-carcinogen factors, and induced genotoxicity and apoptosis in tumoral cells of Swiss mice with DMBA-induced breast cancer^31^.

The essential oil extracted from wood of *Phoebe bournei* (Hemsl.) Yang, which is rich in δ-cadinene, has good antitumor activity against leukemia (HL-60; IC_50_ 42.7 µg/mL), hepatoma (SMMC-7721; IC_50_ 88.1 µg/mL), breast cancer (MCF-7; IC_50_ 54.9 µg/mL), and colon carcinoma (SW480; IC_50_ 75.6 µg/mL) human cell types^32^. These findings help to explain the antitumoral activity of EEOb *in vivo* against ETT showed in this study. Furthermore, the numerous phytochemical constituents in EEOb and their potency to prevent cancer cell growth may be attributed to the additive or synergistic effects of the different components.

The number of AgNOR is related to the cell cycle and proliferative activity of the cell^33^. The quantification of AgNOR area is related to the tumor growth rate, cell proliferative activity and consequently tumor progression^34^^,^ 35. Thus, we used AgNOR quantification to evaluate the antitumor effect of EEOb *in vivo*. In accordance with Fig. 1, the AgNOR number (Control = 2.40 ± 0.31 vs. EEOb = 1.60 ± 0.15; *p* < 0.05) (Figs. 1b and 1c) per nucleus morphometric analysis was significantly higher in the control group compared to animals treated with EEOb (100 mg/Kg). In squamous cell carcinoma of the tongue, there is a correlation between the amount of AgNORs and the degree of differentiation, proliferation rate, and malignancy of the tumor^36^. This correlation was also seen in oral squamous cell carcinoma^37^^,^ 38. Thus, the reduction in the number of AgNORs induced by EEOb suggests that the treatment decreases the rate of cellular proliferation of the EET and impairs its malignity.

**Figure 2:**
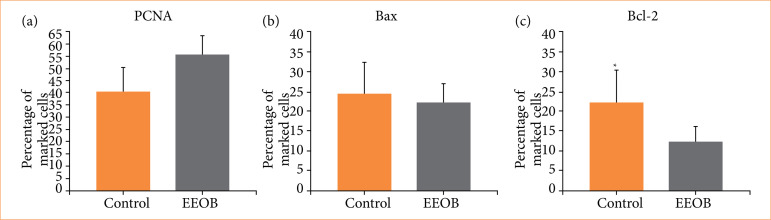
Immunohistochemistry of **(a)** proliferating cell nuclear antigen (PCNA), **(b)** Bax and **(c)** Bcl-2 in mice footpads inoculated with Ehrlich experimental tumor after 28 days and treated with ethanol extract of *Ocimum basilicum* L. (basil) (EEOb) aerial parts. Control group received daily saline solution. EEOb group received daily basil extract at 100 mg/kg intraperitoneally. Data are mean ± standard deviation (n = 6).

Source: Elaborated by the authors. *p < 0.05.

In order to evaluate the possible mechanism of antitumor action of the EEOb, immunohistochemistry for markers of cell proliferation (PCNA) and apoptosis (Bcl-2 and Bax) was performed. Figure 2 shows that the treatment of animals with EEOb at 100 mg/kg was not able to reduce the expression of PCNA (Control = 40.95 ± 6.40% vs. EEOb = 55.64 ± 6.88%; *p* = 0.064) and Bax (Control = 24.50 ± 4.95% vs. EEOb = 22.10 ± 4.33%; *p* = 0.841) from EET. However, the Bcl-2 content is significantly reduced after treatment with the extract (12.48 ± 3.20%) compared to untreated animals (22.57 ± 4.75%; *p* = 0.016). These results shows that EEOb induces apoptosis in EET cells by reducing the content of the anti-apoptotic protein Bcl-2.

Corroborating this study, it was previously demonstrated that the ethanolic extract of *O. basilicum* decreases the expression of the Bcl-2 protein in breast cancer cells (T47D and MCF-7) *in vitro* at concentrations of 199 and 388 µg/ mL, respectively^39^. Moreover, *O. basilicum* extract reverts the increase of Bcl-2 content from testicular tissue induced by acute exposure of cadmium in albino rats, suggesting that the effect of Basil extract on Bcl-2 is maintained in tissues other than breast tissue.

## Conclusion

The results showed that the daily administration of 100 mg/kg of EEOb aerial parts was able to reduce the proliferative speed of EET, evidenced by the reduction of tumor growth and by the smaller number of AgNORs in the tumor cell nuclei.

Furthermore, we showed that this effect may be associated with the ability of *O. basilicum* extract to induce Bcl-2-dependent apoptosis in tumor cells. However, for a more comprehensive understanding of the antitumor mechanism and related effects, further studies are needed to investigate in more detail the cellular signaling pathways involved, the interaction of the extract with other apoptotic proteins, as well as preclinical and clinical studies to evaluate its efficacy and safety in animal and human models. These additional research efforts may provide essential insights for the development of *O. basilicum*-based therapies for cancer treatment.

## Data Availability

Data will be available upon request.
